# Significant correlation between leaf vein length per unit area and stomatal density: evidence from Red Tip and Chinese photinias

**DOI:** 10.3389/fpls.2024.1365449

**Published:** 2024-03-20

**Authors:** Ke He, Karl J. Niklas, Ülo Niinemets, Jinfeng Wang, Yabing Jiao, Peijian Shi

**Affiliations:** ^1^ Architectural Design and Research Institute, Shenzhen University, Shenzhen, China; ^2^ College of Ecology and Environment, Nanjing Forestry University, Nanjing, China; ^3^ School of Integrative Plant Science, Cornell University, Ithaca, NY, United States; ^4^ Institute of Agricultural and Environmental Sciences, Estonian University of Life Sciences, Tartu, Estonia; ^5^ Estonian Academy of Sciences, Tallinn, Estonia

**Keywords:** leaf hydraulic architecture, leaf venation network, *Photinia × fraseri*, *Photinia serratifolia*, regular distribution, spatial repulsion

## Abstract

The vascular veins in photosynthetic leaves play an important role in transporting water and sugars throughout the plant body, and their venation pattern and vein density determine the hydraulic efficiency of the leaf. Likewise, stomatal density (SD) can influence photosynthetic gas exchange. However, the correlation between leaf vein density and SD is seldom reported. Herein, we examined 16 leaves from the hybrid *Photinia* × *fraseri* and 16 leaves from one of its parents, *P. serratifolia*, to explore the correlation between leaf vein density and SD. For each leaf, equidistant lamina quadrats were excised along two longitudinal transects (one along the midrib and another along the leaf margin). For each quadrat, micrographs of 1.2 mm × 0.9 mm stomatal imprints, and 2.51 mm × 1.88 mm micrographs of leaf veins were used to measure total vein area per leaf unit area (VAA) and total vein length per unit area (VLA), as indicators of leaf vein density, to determine the correlation between SD and leaf vein density. For each taxon, there was no significant correlation between SD and VAA, but there was a significant correlation between SD and VLA. The data indicate that SD is not positively correlated with VAA but positively correlated with VLA for both the hybrid and the parent species. This study indicates that future work should focus on the relationships between SD and total vein length per unit area rather than on total leaf vein area per unit area within and across species.

## Introduction

Seed plants have different leaf vein patterns that are traditionally placed into one of four categories: reticulate venation, parallel venation, open dichotomous venation, and single venation (Roth-Nebelsick et al., 2001; Carvalho et al., 2018). The hierarchical reticulate venation is generally considered the most hydraulically complex because of its numerous minor veins, fractal-like organization, and evolutionary recency (Boyce et al., 2009; Carvalho et al., 2018). Leaf vein density has been shown to influence leaf maximum photosynthetic rates by influencing leaf hydraulic efficiency (Brodribb et al., 2007, 2010; Scoffoni et al., 2011). It is usually quantified as the total vein length per unit lamina surface area, which is positively correlated with leaf hydraulic conductance. The quantification of stomatal density (SD, i.e., the number of stomata per unit epidermal area) also plays a vital role in managing the fluxes of carbon dioxide and water vapor and is closely associated with the mean size and geometry of areoles, which are surrounded and defined by different orders of veins, i.e., areoles are the closed polygons formed by different orders of leaf veins (Price et al., 2011; Shi et al., 2022). In turn, the mean distance between stomata and the nearest vein is significantly negatively correlated with SD (Fiorin et al., 2016). In general, the larger the areole, the larger the mean guard cell size within the areole. Given the negative scaling relationship between SD and mean guard cell size (Franks and Beerling, 2009; Liu et al., 2023), a larger areole is consequently associated with a lower SD (Fiorin et al., 2016).

The reticulate venation pattern is thought to be the most effective spatial arrangement of vasculature and stomata to reduce the distance of water transport from veins to stomata. Owing to the reticulate venation pattern and the existence of free-ending veinlets in such a venation pattern, leaf hydraulic resistance is largely reduced, because water transport is more expedient in the xylem pathway than in mesophyll cells (Brodribb et al., 2007). This results in a regular distribution of stomata among areoles due to dense minor leaf veins including free-ending veinlets in some species (Fiorin et al., 2016; Liu et al., 2021; Shi et al., 2021, 2023; Sun et al., 2023). Because of the spatial competition between stomata and leaf veins (Baresch et al., 2019), there exists substantial differences in stomatal size and density across different sizes of areoles (Shi et al., 2021; Sun et al., 2023). Consequently, at spatial scales larger than that of the level of an areole, the extent to which SD and leaf vein density are correlated remains unknown. Stomatal density and leaf vein density are usually listed as two important leaf functional traits that are closely related to leaf hydraulic conductance and gas exchange efficiency (Brodribb and Jordan, 2011; Sack and Scoffoni, 2013). However, in practice, to measure leaf vein density (usually represented by the ratio of total leaf vein length to lamina area) of leaves with hierarchical network venation, it is necessary to chemically clear the mesophyll and epidermal cells. This is very time-consuming. Relative to leaf vein density, SD is faster to measure using the nearest distance between stomata, or by means of a special software using fluorescence micrographs (Liu et al., 2021; Li et al., 2022; Shi et al., 2023). Statistically robust correlations among leaf functional traits, such as leaf vein density and SD, can also provide dependable and efficient methods to predict the relationships among leaf functional traits. Because different metrics for measuring venation complexity have been used by different workers (e.g., vein length versus vein area), we tested the spatial “repulsion” hypothesis, which proposes that stomata are “dispersed” within areoles as a consequence of the locations of veins. If correct, this hypothesis predicts that vein length per unit area will correlate with stomatal density, whereas vein area per unit area will correlate less so, or not at all.

**Figure 1:**
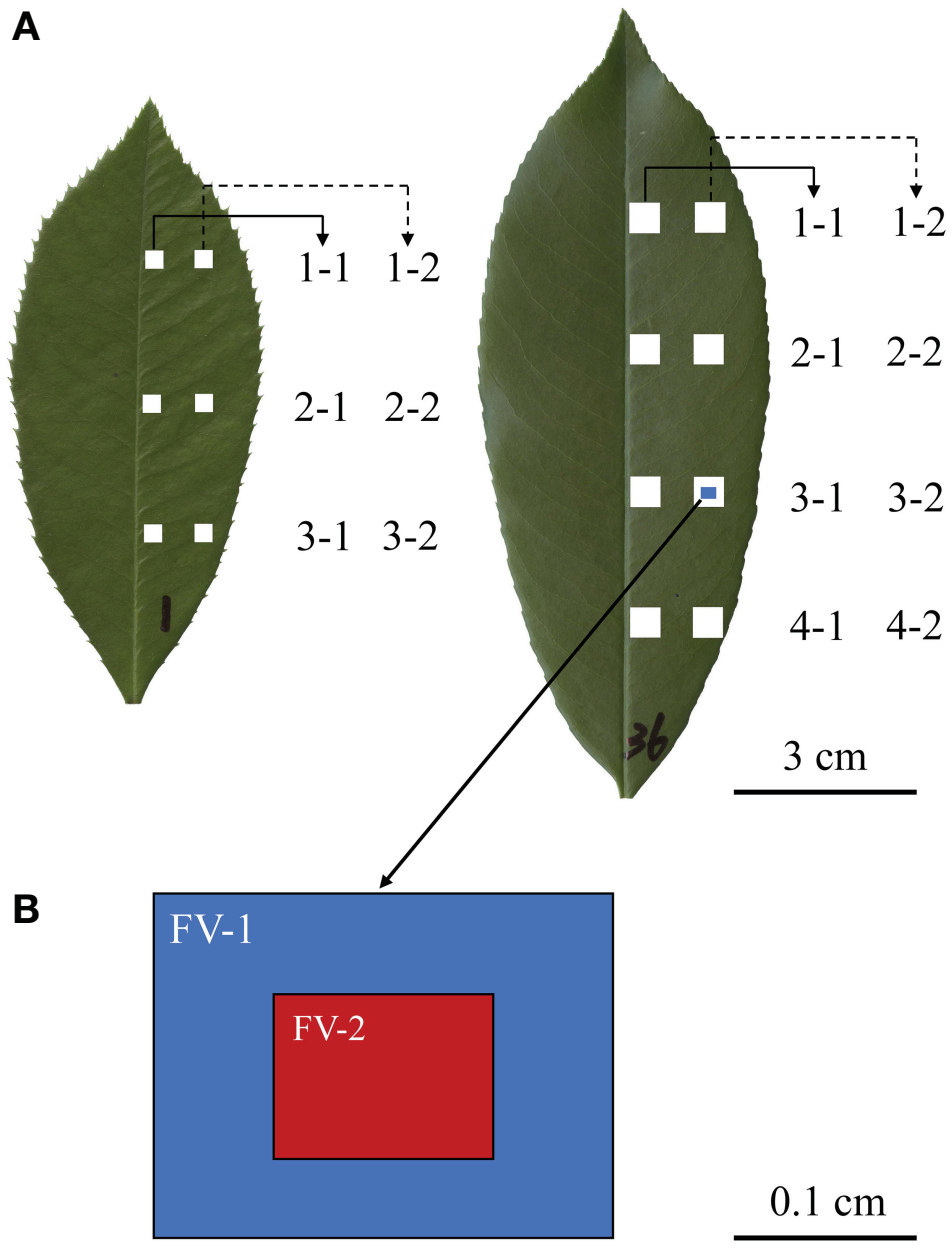
Leaf lamina quadrat locations and numbering scheme for *Photinia × fraseri* and *P. serratifolia* leaves, respectively shown on the left and right of **(A)**, and the notation and relative fields of view for stomata (FV-2) and veins (FV-1) **(B)**. A total of six and eight equally spaced quadrats were removed from each *Photinia × fraseri* leaf and each *P. serratifolia* leaf. Numbers were written on each leaf to distinguish individual leaves from the two taxa for subsequent data analysis. **(B)** shows the size of the leaf vein micrograph (FV-1; 2.51 mm × 1.88 mm) and the size of the stomatal micrograph (FV-2; 1.2 mm × 0.9 mm).

Here, we use a *Photinia* hybrid, called ‘Red Tip’ photinia (*Photinia × fraseri*), and one of its parents, the Chinese photinia (*P. serratifolia* (Desfontaines) Kalkman), to examine and quantify a variety of venation morphometric variables. These taxa were used because their leaves have a typical hierarchical reticulate venation pattern, but differ substantially in size (Zheng et al., 2022; **Figure 1**). Sixteen leaves from each of the two taxa were used, with six 0.3 cm *×* 0.3 cm lamina quadrats for each *P. × fraseri* lamina and eight 0.5 cm *×* 0.5 cm lamina quadrats for each *P. serratifolia* lamina. For each quadrat, one 2.51 mm × 1.88 mm leaf-vein micrograph and one 1.2 mm × 0.9 mm stomatal micrograph within the leaf-vein micrograph were used to calculate total leaf vein area per unit area (VAA) and total vein length per unit area (VLA) based on the one leaf-vein micrograph, and to calculate SD based on the one stomatal micrograph. This protocol generated 96 and 128 VAA, VLA and SD values for the two taxa, respectively. The goal was to (i) quantify VAA, and VLA along two longitudinal transects along the same side of the lamina, and to determine (ii) whether there are significant correlations between SD and VAA and between SD and VLA. We did not compare the second parent (*Photinia glabra* (Thunb.) Maxim.) with the hybrid because the second parent did not differ in leaf size compared to the first parent and because a leaf-size difference between any two taxa was a key factor in testing our hypothesis. Specifically, the hybrid ‘Red Tip’ has a much smaller lamina size compared to the one parent *P. serratifolia* (Zheng et al., 2022).

## Materials and methods

### Plant growth sites and leaf sampling

Thirty mature and undamaged leaves were randomly sampled from the middle canopies of ten *P. × fraseri* individuals at one site (118°48'35'' E, 32°04'67'' N), and another thirty mature leaves were sampled from three *P. serratifolia* individuals at another site (118°48′37″ E, 32°04′45″ N) both in Nanjing Forestry University Campus, on 15th July, 2022. In mid-July, leaves matured such that leaf vein and stomatal traits reached completion and could be accurately measured. Nanjing has a humid subtropical climate, which is influenced by the East Asian Monsoon. The mean annual precipitation and mean annual temperature of Nanjing are 1156 mm and 15.6°C, respectively, based on the climate data recorded between 1951 and 2014 (Jiao et al., 2022). The rainy season is concentrated from June to August, and the total precipitation of the three months account for approximately 50% of the annual precipitation of Nanjing. In a strictest sense, *P. × fraseri* is a hybrid of *P. serratifolia* and *P. glabra*. However, henceforth *P. × fraseri* is referred to as a ‘species’ for simplicity. For each species, we obtained intact micrographs of stomata and leaf veins from 16 leaves for each taxon (see below for details). The 14 leaves for each taxon were discarded because leaf veins of some lamina sections on these leaves were not successfully obtained during removing mesophyll cells (see below for details).

### Lamina sampling and micrographs of stomata and leaf veins

For each leaf, equidistant lamina quadrats (three 0.3 cm *×* 0.3 cm for *P. × fraseri* and four 0.5 cm *×* 0.5 cm for *P. serratifolia*) were excised along two longitudinal transects (one along the midrib and another along the leaf margin; see **Figure 1**). The difference in the size of lamina quadrats reflects the difference in the size of the lamina area between the two taxa. *P. serratifolia* has larger leaves, and leaf veins are less easy to damage when sampling a larger lamina quadrat. To examine if differences in the variables of interest existed from the midrib to the leaf margin, two longitudinal transects both on the right side of the lamina were studied (**Figure 1**).

All quadrats were painted with colorless nail polish to obtain stomatal imprints. Stomatal imprints were viewed with a Leica DM 2500 optical microscope (Leica Microsystems Shanghai, Shanghai, China) with a magnification of 10 × 10 and stomatal micrographs (each measuring 1.2 mm × 0.9 mm in area) were taken using LAS X software (version 3.4.2.18368; Leica Microsystems CMS GmbH, Germany), approximately in the center of each lamina quadrat, and saved as TIF files.

**Figure 2:**
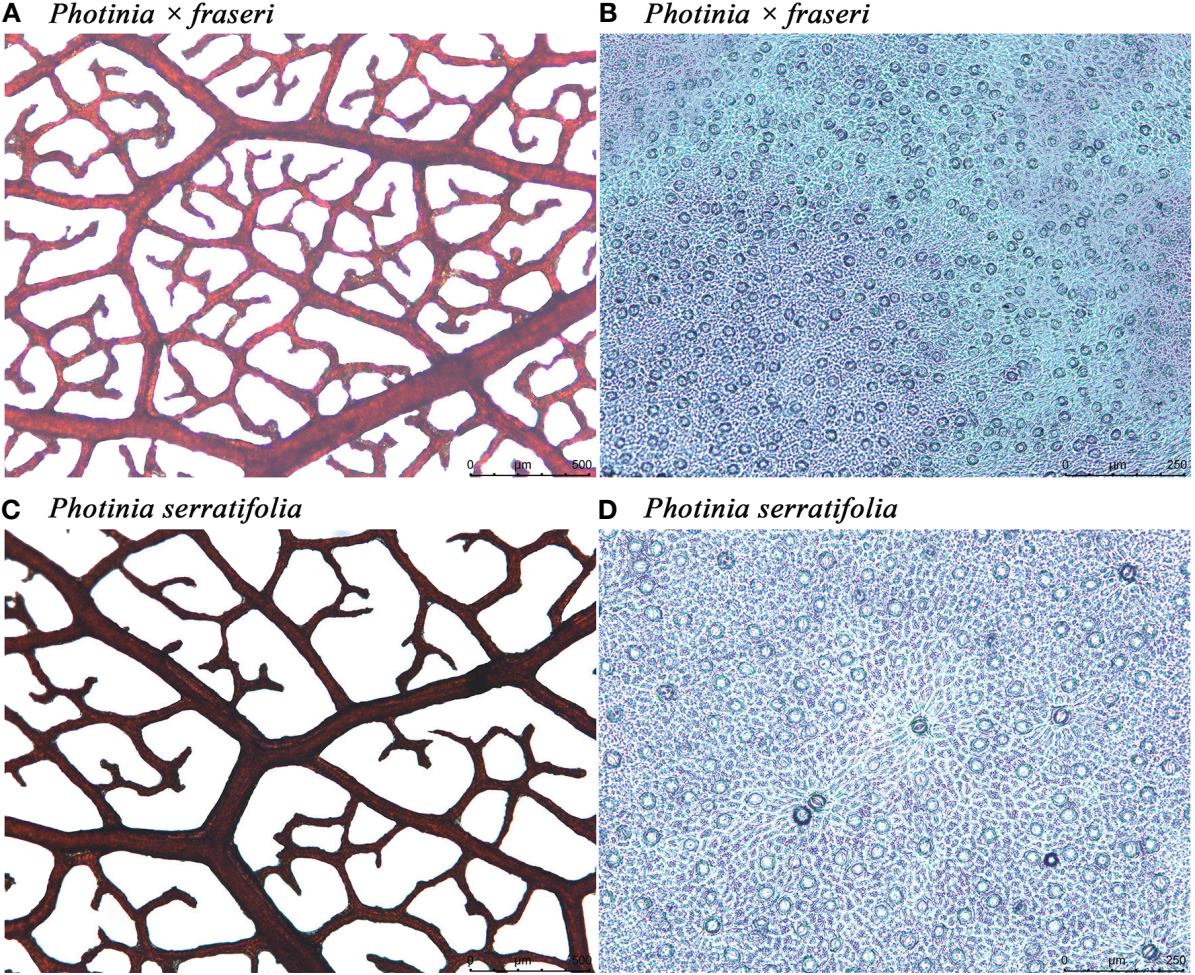
Leaf vein and stomatal micrographs of *Photinia × fraseri* **(A, B)** and *P. serratifolia* **(C, D)**. In **(A, C)**, the size of each leaf-vein micrograph is 2.51 mm × 1.88 mm, and in **(B, D)**, the size of each stomatal micrograph is 1.2 mm × 0.9 mm. The colorless nail polish approach did not provide clear profiles of guard cells. In fact, the elliptical geometries are all stomata because there were no cross-sections of fuzzes on the leaf surface. When enlarging the image in counting the number of stomata, we can roughly see the profiles of guard cells.

Each quadrat was transferred into a 10% NaOH solution to digest and remove mesophyll cells after 2 to 3 weeks of submersion. Residual mesophyll cells were subsequently removed by wiping by hand the interior of the epidermis along the direction of the veins. The remaining tissues were then washed with water, stained with a 0.5% w/v aqueous safranin solution, and viewed with the Leica optical microscope with a magnification of 10 × 5. Micrographs (2.51 mm × 1.88 mm) were taken with the Leica microscope camera using LAS X software aimed approximately in the center of each lamina quadrat and saved as TIF files (see **Figures 1**, **2** for details). The size of stomatal micrographs is approximately 1/4 that of leaf vein micrographs.

### Estimation of stomatal density and leaf vein density

The micrographs of leaf veins were converted from RGB to a grayscale using Photoshop (version 13; Adobe Systems Incorporated, San Jose, CA, USA). The “readTIFF” function of the “tiff” package (version 0.1-11; Urbanek and Johnson, 2022) in R (version 4.2.0; R Core Team, 2022) was used to read each leaf-vein micrograph. The pixel values in an image ranged from 0 to 255, where 0 represents black and 255 represents white. A critical value of 200 was set to distinguish the pixels associated with leaf veins when the values of pixels of leaf vein micrographs were smaller than the critical value. The remaining pixels were the areas occupied by areoles. The leaf vein area per unit area (VAA) was calculated as the number of leaf vein pixels divided by the number of the total pixels per micrograph measuring 2.51 mm × 1.88 mm. The LEAF GUI software (Price et al., 2011) based on MATLAB (version R2016b; MathWorks, Natick, MA, USA) was used to calculate the leaf vein length per unit area (VLA) for each leaf vein micrograph. The leaf vein density is the leaf vein length per unit area (mm/mm^2^).

### Statistical analysis

A linear mixed-effects model (Pinheiro and Bates, 2000) was used to test for the significance of the differences in VAA and VLA between any two leaf-vein micrographs of the same species. The positions of leaf-vein micrographs on leaves were used as categorical fixed effects and leaves as random effects. The SD data have been analyzed in Sun et al. (2023) and are not repeated in the present study. Correlation tests at the 0.05 significance level were carried out to examine the relationships between SD and VAA, and between SD and VLA. The data of SD, VAA and VLA can be accessed in online **Supplementary Table S1**. The statistical software R (version 4.2.0; R Core Team, 2022) was used to perform correlation analyses, and a specialized R package “nlme” (version 3.1-157; Pinheiro and Bates, 2000) was used to perform the linear mixed-effects model.

## Results

**Figure 3:**
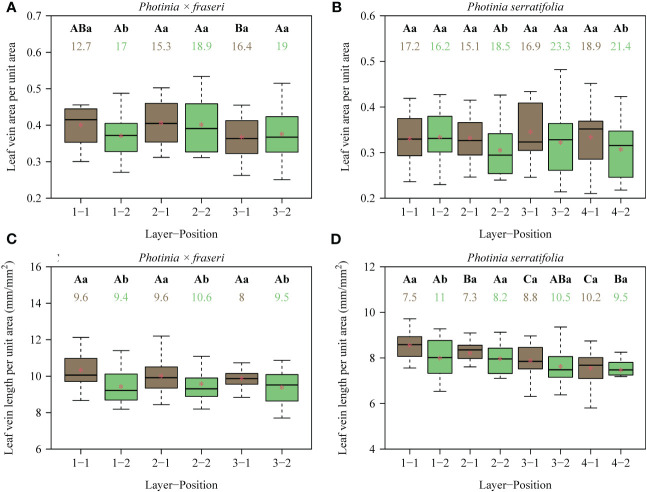
Box-and-whisker plots for the leaf vein area per unit area **(A, B)**, and the leaf vein length per unit area **(C, D)** for each position of each layer (see **Figure 1** for the numerical code for each position; *n* = 16 for each position). Uppercase letters at the top of the whiskers indicate the significance of differences between any two layers (from the leaf apex to leaf base) based on a linear mixed-effects model with the leaf number as a random effect. Lowercase letters show the significance of the difference between any two positions (leaf midrib vs. right leaf margin, **Figure 1**) based on a linear mixed-effects model with the leaf number as a random effect. The numbers below the letters are the coefficients of variation (%) of vein area per unit area or vein length per unit area. The segments in the boxes represent the medians, and the asterisks near the segments represent the means. On the *x*-axis in the figure, the number before the hyphen is the layer number from the leaf apex to the leaf base; the number after the hyphen is the longitudinal transect number from the midrib to the right leaf margin. Because the lamina of *P. × fraseri* is smaller than that of *P. serratifolia*, there were only three layers from the leaf apex to leaf base. In contrast, the lamina of *P. serratifolia* is sufficiently large that four layers from the leaf apex to leaf base could be studied (see **Figure 1** for details). Thus, in **(A, C)** (representing *P. × fraseri*), the maximum number before the hyphen is 3, and in **(B, D)** (representing *P. serratifolia*), the maximum number before the hyphen is 4.

**Figure 4:**
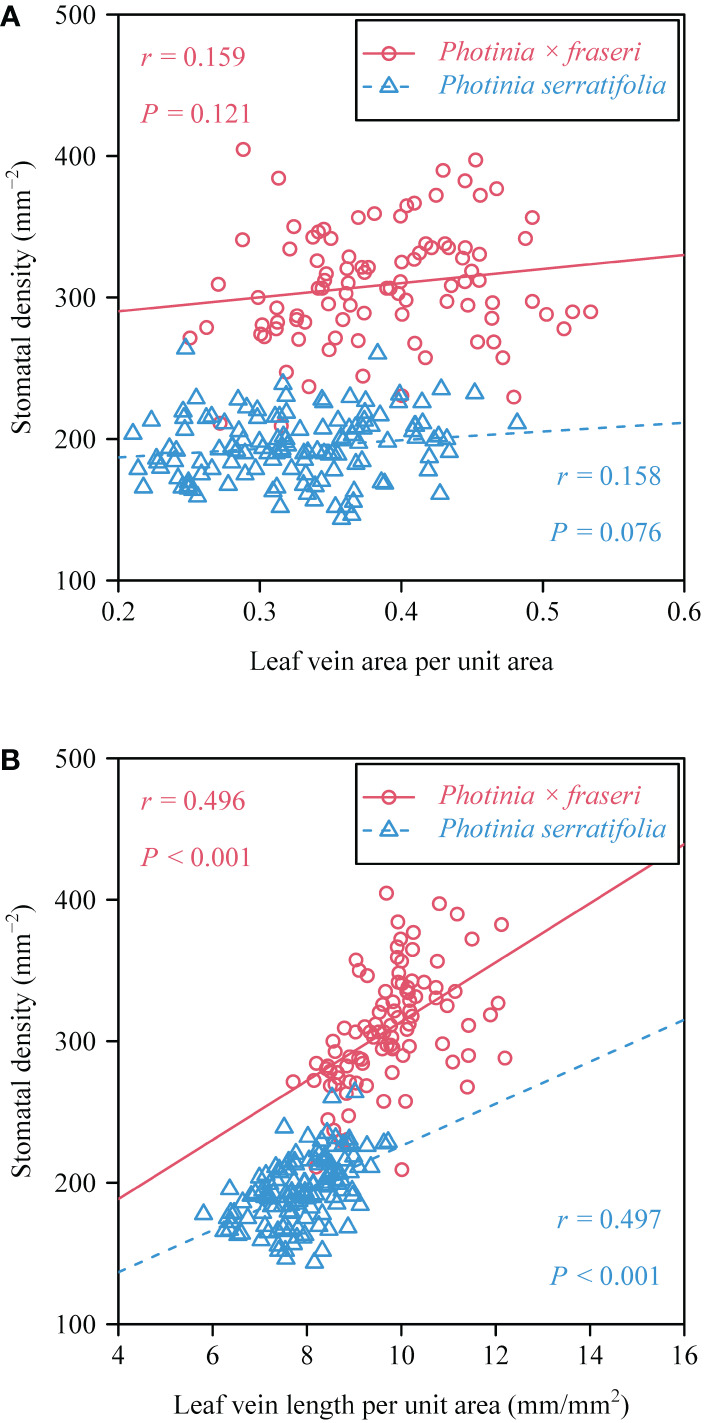
Bivariate plots and ordinary least squares correlation statistics for stomatal density vs. the leaf vein area per unit area **(A)**, and stomatal density vs. leaf vein length per unit area **(B)**. In each panel, the open circles and triangles represent the observations of the two taxa (see upper right inserts for symbols of the taxa), and the straight lines are the regression lines; *r* is the correlation coefficient; and *P* represents the *P*-value of the correlations test.

For the 16 *Photinia × fraseri* leaves, the numerical values of total leaf vein area per unit area (VAA) ranged between 0.25 and 0.53, and those of total leaf vein length per unit area (VLA) ranged between 7.7 and 12.2 mm/mm^2^ (**Figures 3A, C**). For the 16 P*. serratifolia* leaves, the numerical values of VAA ranged between 0.21 and 0.48, and those of VLA ranged between 5.8 and 9.7 mm/mm^2^ (**Figures 3B, D**). By comparison with the medians of VAA and VLA along the transect close to the leaf margin, the medians of VAA and VLA along the transect close to the midrib tended to be greater (**Figure 3**). However, the lateral (midrib-to-leaf margin) differences along some transects were not statistically significant apart from the values of VLA of *P. × fraseri* (**Figure 3C**). There were no significant trends in VAA basipetally from the leaf apex to the leaf base. Nevertheless, VLA tended to decrease basipetally from the leaf apex to the leaf base along the longitudinal transect close to the midrib and from the midrib to the right leaf margin (**Figures 3C, D**). There was no significant correlation between stomatal density (SD) and the VAA (*r* = 0.159 and *P* > 0.05 for *P. × fraseri*; *r* = 0.158 and *P* > 0.05 for *P. serratifolia*; **Figure 4A**), but there was a significant correlation between SD and VLA for each taxon (*r* = 0.496 and *P* < 0.05 for *P. × fraseri*; *r* = 0.497 and *P* < 0.05 for *P. serratifolia*; **Figure 4B**).

## Discussion and conclusions

Prior work with species producing leaves with hierarchical reticulate venation has shown that stomatal density (SD) exhibits a significant spatial variation across the leaf lamina across areoles, while the spatial arrangement of stomata tends to be regularly dispersed due to the dense reticulate venation network at the areole level, i.e., there is spatial “repulsion” between stomata at the areole level (Liu et al., 2021; Shi et al., 2021, 2023). Against this overall backdrop, the data presented here based on a hybrid and one of its parents indicate that the total leaf vein area per unit area (VAA) does not show a linear variation in the leaf longitudinal orientation, whereas the total leaf vein length per unit area (VLA) decreases basipetally from the leaf apex to leaf base, and from the midrib to leaf margin. There is no statistically significant correlation between SD and VAA, in contrast to a significant correlation between SD and VLA, which has been proposed as a useful measure of leaf vein density by Sack and Scoffoni (2013). At the whole leaf level, total leaf vein length is found to be proportional to total leaf vein area (Shi et al., 2022). Nevertheless, this proportionality does not indicate that VLA is proportional to VAA when viewed across small spatial scales such as the size of the micrographs used in this study (see **Figure 1B**). Mediavilla et al. (2020) report that SD does not significantly correlate with each of the leaf vein measures including total leaf vein density, total major vein density, or total minor vein density for each of three *Quercus* species (*Quercus faginea* Lam., *Q. suber* L., and *Q. ilex* L. subsp. *ballota* (Desf.) Samp.). However, their micrographs of stomata do not overlap with the micrographs of leaf veins (two leaves for measuring SD and another two leaves for measuring leaf vein traits were used) in their study. Failure to detect a correlation between SD and leaf vein density (as measured by VLA) as reported by Mediavilla et al. (2020) might result from the spatial variation in SD and VLA across different positions within the same leaf and across leaves.

The overall coordinated spacing of leaf veins and stomata is hypothesized to be an adaptation that maximizes the hydraulic and gas-exchange functionality of the leaf lamina, as suggested in part by the observation that mean SD is linearly related to the length of the vein contours around areoles (Fanourakis et al., 2015; Fiorin et al., 2016). The data presented here indicate that future work should focus on the mean distance from stomatal centers to the nearest veins including free-ending veinlets, and model the scaling relationship between SD and the mean distance between veins and evaporative sites across closely related species, and comparing the scaling exponents at the intra- and interspecific levels, rather than only focusing on the correlation between SD and VLA.

In summary, using micrographs of two uniform sizes, we examined whether there were significant correlations between SD and both VAA and VLA as metrics reflecting leaf vein density. The data failed to reveal a correlation between SD and VAA, but indicted a significant correlation between SD and VLA. This work suggests that future studies should focus on the relationships between SD and VLA rather than on VAA within and across species.

## Data availability statement

The original contributions presented in the study are included in the article/**Supplementary Material**. Further inquiries can be directed to the corresponding authors.

## Author contributions

KH: Formal analysis, Writing – original draft. KN: Formal analysis, Writing – review & editing. ÜN: Formal analysis, Writing – review & editing. JW: Investigation, Writing – review & editing. YJ: Investigation, Writing – review & editing. PS: Formal Analysis, Methodology, Writing – review & editing.
